# The Medical Profession and Public Holidays

**Published:** 1914-12-26

**Authors:** 


### The Hospital

The Workers' Newspaper of Administrative Medicine and Institutional Life, Administration, National Insurance and Health.

No. 1488, Vol. LVII. SATURDAY, DECEMBER 26, 1914. PRICE ONE PENNY.

### THE MEDICAL PROFESSION AND PUBLIC HOLIDAYS.

Members of the medical profession are able for the most part to survey the public holiday with a detached and impartial eye. They rarely take much part in these festivities, for the claims of the sick and suffering cannot be relaxed by Acts of Parliament. Hence when the world and his wife are taking holiday and making merry the doctor must continue his daily duty and task. In his secret soul he probably is rather glad than sorry on this account. He may, in words, contrast his hard lot with that of his freer neighbours, but, granted only that his heart is in his work, he is not really displeased that his obligation should continue, though the rest of the world is at play. After all, his training, if not his disposition, fits him for such experiences. Set hours and fixed times have never been his environment, and he is unaccustomed to "down tools" at particular days and hours. In all this there are no doubt hardships, but there are also compensations, and most of us are more than willing to put up with the one for the sake of the other. Perhaps of all holidays the one which carries the most trying experiences for the doctor is the season of Christmas, and the man who is summoned from the family dinner-table to a midwifery case and yet is able to depart with goodwill, has a claim on the attention of the most stoic of philosophers. If such a summons is awaiting any one of our readers he will have a great opportunity for the equal mind. The sympathy of his family and the gratitude of his patient may help to sustain him, but he must rely mainly on his own resolution. In any event, we wish both him and those to whom the times are more propitious all the goodwill appropriate to the season. Should the demand of duty clash with other claims, we have no doubt which will carry the day.

The public holiday has to the profession other aspects than that of personal convenience. It sometimes brings in its train a series of sufferers, by no means solely of the youthful order. Activities, digestive and otherwise, are apt to be strained on these occasions, and the sequel may be a painful one. Possibly such a prospect may be helpful to the doctor's philosophy. All the same he will not hesitate to give good advice towards the prevention of these minor disasters. Sudden and severe changes, whatever be the direction they take, put a strain on the bodily machinery, and this especially when the machine has lost the elasticity of its first youth. Hence holiday-makers may be warned in advance to take their pleasures wisely and not too well; and the advice may be emphasised in relation to those for whom anniversaries are occasions rather for reminiscence than for anticipation. Such are the dictates of physiological truth, and Nature knows no occasion for the relaxation of her bond. Yet the public holiday is not an opportunity for the perpetual reiteration of the things which are forbidden. Excess, even in prudence, is possible, and man is not made for the anxious contemplation of all the risks which peradventure may befall him. So melancholy a philosophy would throw gloom on all our activities, and the event would not seldom prove that the anxiety had been needless. The old gentleman who, towards the close of his life, remarked that he had had many troubles and most of them hadn't happened, summarised a not infrequent experience, and both he and Mrs. Gummidge may be quoted to those who are overcharged with good advice. There is a time for most things, if not for all, and the holiday, with its detachment from routine and its excursion into the exceptional, has a sound physiological basis, and one well established in human experience.

Perhaps the direction in which the Christmas holiday excites special anxiety is that of eating and drinking. The dangers which lurk in the wine-cup are set forth from certain quarters in lurid terms, and an analytical contemplation of the mince-pie and the plum-pudding produces warnings which might well dismay the stoutest appetite. Other foodstuffs, with nuts and cheese in the foreground, offer, on the other hand, all the attractions of delicately adjusted proximate principles exactly suited to the needs of the human economy. Yet, curiously enough, the world gives but scanty heed to these repeated counsels. In vain do the prophets of the laboratory announce innumerable woes as the consequences of a diet which fails to satisfy their chemical tests. Good cheer continues to make its successful annual appeal, and apparently a blind and wilful public rushes on its fate. It is not necessary to state here that the medical profession is no advocate of excess in eating and drinking. The question may, however, be fairly asked whether the dietetic customs of the Christmas season are so foolish as some would paint them. After all, while man is an animal, he is the most intelligent of animals, and a choice which is not challenged in those of lower rank cannot be wholly without wisdom in the head of the series. Habit and custom, from the very fact that they exist, have at least a *prima facie* justification, and must have some root in experience. It is possible, further, to take too restricted a view of the purposes of eating and drinking and to judge them solely as factors in tissue nutrition. This is to lose sight of their mental and moral values, which, at least as we think, are considerable. Towards such ends we suggest that Christmas customs make some contribution, and we believe they can be defended by sound physiological reasons. If any of our readers are disposed to challenge this proposition we shall be prepared to defend it when the Christmas of 1914 is numbered with the past. In the meantime may "good digestion wait on appetite, and health on both."

